# Selective improvements in balancing associated with offline periods of spaced training

**DOI:** 10.1038/s41598-018-26228-4

**Published:** 2018-05-18

**Authors:** Antonino Casabona, Maria Stella Valle, Carlo Cavallaro, Gabriele Castorina, Matteo Cioni

**Affiliations:** 1 Neuro-Biomechanics Laboratory, Department of Biomedical and Biotechnological Sciences, University of Catania, 95123 Catania, Italy; 2 Physical Medicine and Rehabilitation Residency Program, University of Catania, 95123 Catania, Italy; 3 Gait and Posture Laboratory, Policlinico-Vittorio Emanuele Hospital, 95123 Catania, Italy

## Abstract

Benefits from post-training memory processing have been observed in learning many procedural skills. Here, we show that appropriate offline periods produce a performance gain during learning to stand on a multiaxial balance board. The tilt angle and the area of sway motion of the board were much more reduced in participants performing a training spaced by an interval of one day with respect to participants executing the same amount of practice over a concentrated period. In particular, offline memory encoding was specifically associated with the motion along the anterior-posterior direction, the spatio-temporal dynamics, and the frequency contents of the board sway. Overall, quantification of spaced learning in a whole-body postural task reveals that offline memory processes enhance the performance by encoding single movement components. From a practical perspective, we believe that the amount of practice and the length of inter-session interval, adopted in this study, may provide objective insights to develop appropriate programs of postural training.

## Introduction

Learning experience based on trial-by-trial movement repetition is marked by an early stage with a fast performance improvement, followed by a more gradual gain as the practice continues.

Learning a new motor skill does not finish with the period of practice. The elaboration of information acquired during sessions of practice continues offline after training, determining further performance improvements and additional long-term memory consolidation.

Thus, within-session changes (online) are associated with the movement repetition, while the between-session gain (offline) depends on memory traces developed during the pauses of training.

The between-session effects can be showed spacing the training with intervals of appropriate length^1–4^. The presence of offline memory processing has been reported in studies ranging from cellular to system level, and several factors have been individuated as determinant to accomplish this process^5,6^. The length of time after training^3,7,8^, the amount of practice^9,10^, sleep intervals^10–12^, conditions of task execution^8,13,14^, and interferences from other tasks^15^, are the most common elements activating and/or modulating the effects of spaced training on learning motor skills.

These effects have been studied mostly in movement paradigms, such as finger sequence tapping^2,16–18^, visuomotor skills^19,20^, reaching in force fields^4,21^, while more commons everyday gross motor skills, such as walking or upright standing, have remained poorly explored^22–24^.

Since it is not obvious that results from single limbs movements can be transferred to whole-body actions^25^, it may be important to test whether benefits from offline learning can be also observed in gross motor abilities. This would contribute to support the idea that general rules might guide motor memory formation across diverse tasks. Moreover, given that movement properties may evolve independently of each other over the stages of learning^4,18^, motor skills that require multiple sensory inputs and multi-joint coordination may offer the opportunity to dissociate motor components and to explore possible specific relationships with online/offline learning.

From an ecological view, the use of tasks with an important impact on the real life would allow to estimate the utility of spaced training in fields such as rehabilitation, sport sciences and occupational physiology.

With this perspective in mind, we studied the effects of spaced training on learning upright standing on a multiaxial balance board.

In a previous paper, we showed that healthy young adults were able to stabilize a multiaxial balance board with a short time of practice and to maintain the acquired skill after one week^26^. During the learning time course, the overall postural stability progressively improved across the sessions of practice, but changes in spatio-temporal dynamics and in the spectral contents of the postural oscillations occurred specifically along the anterior-posterior (AP) direction and only one week after the end of the training. Typically, the AP and medial-lateral (ML) directional components exhibit functional differences also during quiet stance, with more unstable movements along the AP than ML direction^27–29^.

Starting from these data, two main working hypotheses were tested: first, we asked whether the amount of online practice adopted in the previous study could produce further improvements of the postural performance when the sessions were separated by an interval of one day; second, to take advantage from the multiple sensory and motor elements required to accomplish a challenging balance task, we explored the hypothesis that some components of postural control could be specifically influenced by the between-session pause rather than the online practice.

In particular, the comprehensive parameterization implemented in the current study allowed to discriminate between the motion along the AP and ML direction and between changes in performance stability and spatio-temporal structure of the sway oscillations. Performance stability, i.e the ability to keep horizontal the balance board, can be measured by parameters such as amplitude and variability of board tilt angle or area and length of the trajectory traced by board motion (this set of parameters is indicated as ‘stability-related parameters’). An appropriate level of postural stability can be obtained independently from the type of temporal, frequency and spatial structure of the sway oscillations. Spectral analysis and non-linear computations of sway oscillations signals can be used to estimate frequency distribution and temporal structure, while the spatial structure can be assessed computing the fractal dimensionality of the board motion trajectory. This set of parameters is indicated as ‘structure-related parameters’ and includes the Mean Power Frequency (MPF), the Approximate Entropy (ApEn) and the Fractal Dimension (FD). These techniques are increasingly used to capture the complexity of temporal and spatial organization of motor control and to bring out subtle components of motor learning^30^.

## Results

**Figure 1:**
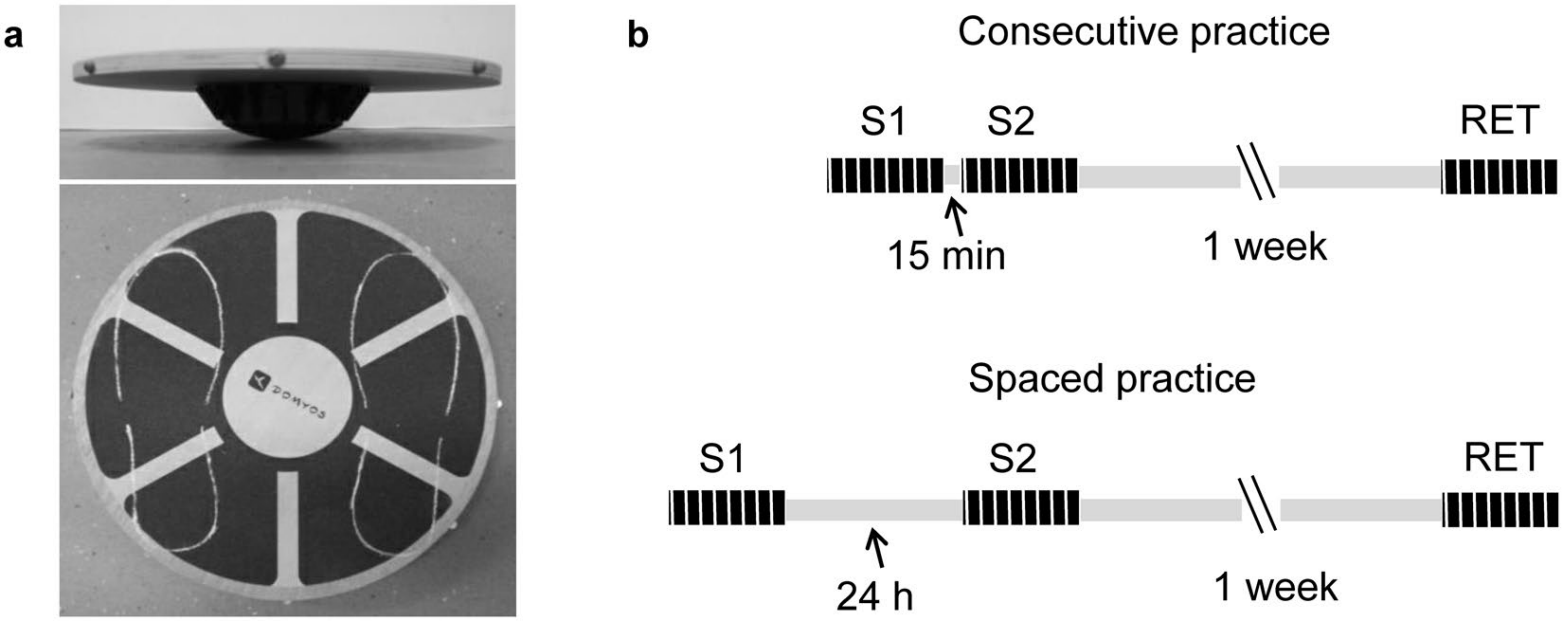
Experimental set up. (**a**) Multiaxial balance board used in the experimental protocol. (**b**) Organization of learning sessions. Two training sessions (S1, S2) were interspersed by 15 min in the consecutive practice and by 24 h in the spaced practice. A retention (RET) session was performed 1 week later. Each session included eight trials of balancing of 20 sec duration.

Two groups of healthy subjects performed three sessions of balancing exercise with a multiaxial balance board (Fig. 1a). Two sessions of practice (S1, S2) and one retention session (RET) after one week were executed (Fig. 1b). One group addressed the two practice sessions with an inter-session pause of 15 min (consecutive practice, CP), while the other group performed the training with an inter-session pause of 24 h (spaced practice, SP).

**Figure 2:**
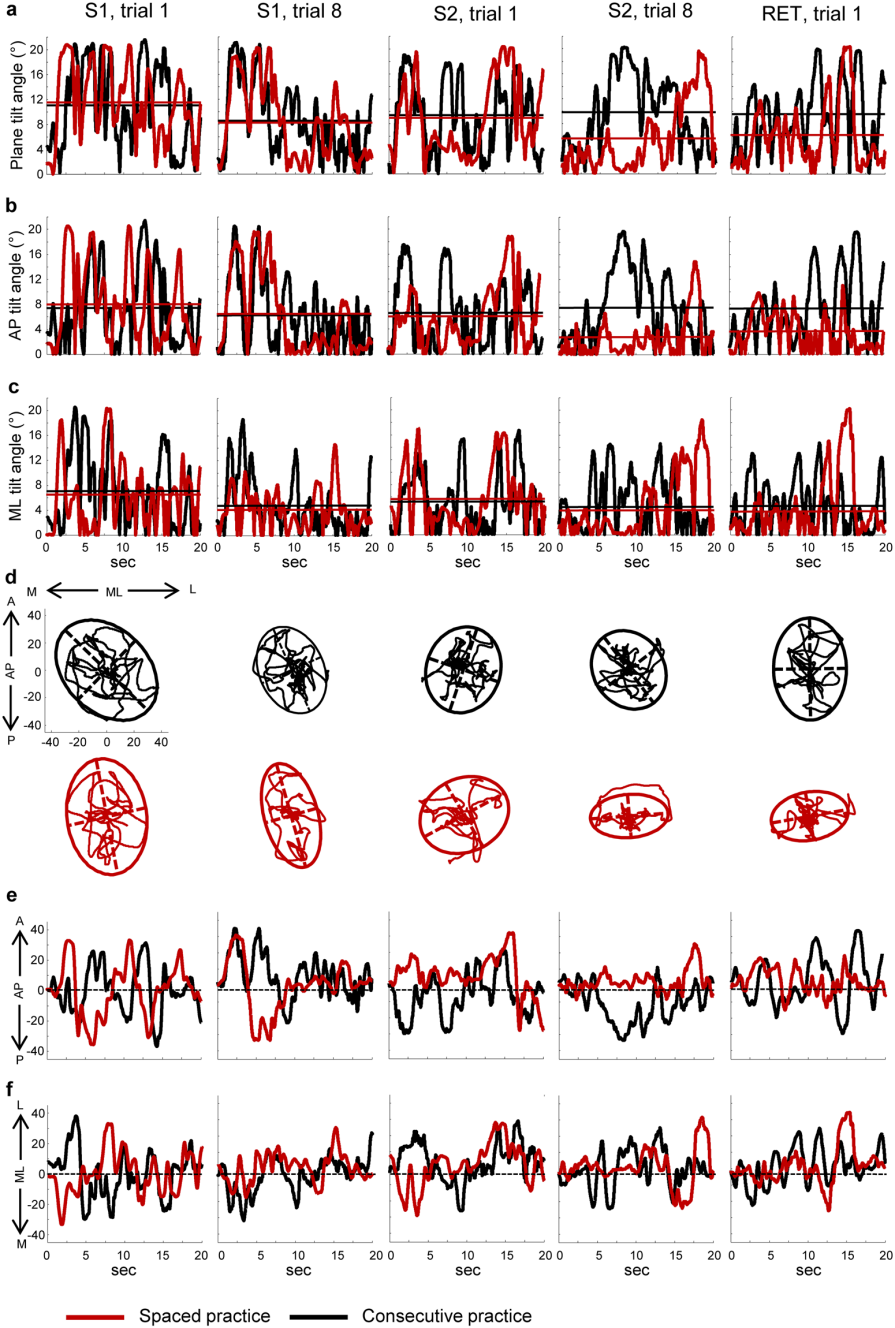
Examples of postural performance measurements. The plots show data from single trials recorded in a subject performing consecutive practice (black lines) and a subject performing spaced practice (red lines). The motion of the balance board was measured during the first and the last trial of the first session (S1), the first and the last trial of the second session (S2) and the first trial of the retention session (RET). Changes in tilt angle amplitude are reported for board movements over any direction (**a**), along the AP (**b**) and ML (**c**) direction. Horizontal lines represent the average value for each time series. The motion of the balance board upon the horizontal plane was quantified by tracking the two-dimensional trajectory of the board normal vector (**d**). From these data points were computed the total sway area (95% confidence ellipses in d), the total sway path and the time series associated with the AP (**e**) and the ML (**f**) direction. The values of variability (RMS and ApEn) and frequency domain (MPF) associated with the time series in e and f are reported in the Supplementary Table S1. The axes units reported in the plots showed in d and in vertical axes of the plots showed in e and f derive from measurements of the space as unit normal vector.

Two representative examples of the overall learning and the effects of the practice conditions are illustrated in Fig. 2. The measurements were recorded from single trials in a participant performing CP (black lines) and in a participant performing SP (red lines). Decreases in the plane tilt angle of balance board (the absolute value of the tilt angle along any direction; Fig. 2a), in the axis tilt angle (the absolute value of the tilt angle along the AP or ML directions; Fig. 2b–c) and in spatial (Fig. 2d) and temporal (Fig. 2e,f) motion of the board normal vector, were observed passing from the first to the last trial of the S1 for both participants. Within the S2 the performance was unchanged for the participant performing the CP, but the subject performing the SP showed important improvements from the first to the last trial for all the parameters, except for the tilt angle amplitude and the sway variability along the ML direction (Fig. 2c,f). The differences between the two subjects were maintained in the first trial recorded during the RET session.

The scheme depicted in these examples is replicated in the summary of results for two-dimensional parameters, i.e. the parameters measuring the board motion related to the horizontal plane (three-way ANOVA with group, session, trial and their interactions as factors; Table 1), and for one-dimensional parameters, i.e the parameters measuring the board motion along the AP or ML directions (four-way ANOVA, with group, session, trial, direction and their interactions as factors; Table 2). After the quantification of changes over the entire time-course of learning and memory retention (S1, S2 and RET), the effects of spaced training were evaluated comparing S1 with S2, while the level of the performance after one week from the end of practice was assessed comparing the S2 with RET.

**Table 1:** Summary of ANOVAs for the two-dimensional parameters.

S1 vs S2 vs RET		1. G	2. S	3. T	4. G x S	5. G xT	6. S x T	7. G x S x T
		df: 1, 18	df: 2, 36	df: 7, 126	df: 2, 36	df: 7, 126	df: 14, 252	df: 14, 252
1. **Total angle**	F	3.783	38.306	15.961	3.109	1.452	3.334	0.737
	P	0.071	**<0.001**	**<0.001**	0.081	0.22	**0.002**	0.648
	η_p_^2^		**0.68**	**0.47**			**0.16**	
2. **Area**	F	2.779	73.471	20.41	1.147	0.869	5.808	0.781
	P	0.116	**<0.001**	**<0.001**	0.319	0.497	**<0.001**	0.601
	η_p_^2^		**0.80**	**0.53**			**0.24**	
3. **Sway Path**	F	0.024	23.953	15.586	1.3	0.876	3.926	0.544
	P	0.879	**<0.001**	**<0.001**	0.283	0.485	**0.001**	0.784
	η_p_^2^		**0.57**	**0.46**			**0.18**	
4. **FD**	F	1.576	6.988	1.105	3.13	0.525	0.741	0.63
	P	0.229	**0.004**	0.36	0.062	0.691	0.608	0.694
	η_p_^2^		**0.28**					
**S1 vs S2**		**df: 1, 18**	**df: 1, 18**	**df: 7, 126**	**df: 1, 18**	**df: 7, 126**	**df: 7, 126**	**df: 7, 126**
5. **Total angle**	F	2.976	87.125	16.188	12.1	1.037	5.154	0.869
	P	0.105	**<0.001**	**<0.001**	**0.003**	0.397	**0.001**	0.498
	η_p_^2^		**0.80**	**0.47**	**0.35**		**0.22**	
6. **Area**	F	2.929	147.268	25.663	4.837	0.668	8.12	0.971
	P	0.108	**<0.001**	**<0.001**	**0.044**	0.638	**<0.001**	0.429
	η_p_^2^		**0.87**	**0.59**	**0.18**		**0.31**	
7. **Sway Path**	F	0.107	45.786	20.053	0.666	0.715	4.95	0.486
	P	0.748	**<0.001**	**<0.001**	0.427	0.659	**0.001**	0.752
	η_p_^2^		**0.68**	**0.53**			**0.22**	
8. **FD**	F	1.092	5.596	0.406	4.586	0.248	0.811	0.339
	P	0.313	**0.032**	0.735	**0.049**	0.848	0.523	0.85
	η_p_^2^		**0.20**		**0.17**			
**S2 vs RET**		**df: 1, 18**	**df: 1, 18**	**df: 7, 126**	**df: 1, 18**	**df: 7, 126**	**df: 7, 126**	**df: 7, 126**
9. **Total angle**	F	5.63	7.771	6.47	0.033	2.04	1.249	0.497
	P	**0.031**	**0.014**	**<0.001**	0.858	0.057	0.296	0.772
	η_p_^2^	**0.20**	**0.26**	**0.26**				
10. **Area**	F	3.643	13.873	6.512	1.112	1.552	1.065	0.472
	P	0.076	**0.002**	**<0.001**	0.308	0.203	0.382	0.759
	η_p_^2^		**0.39**	**0.27**				
11. **Sway Path**	F	0.214	5.11	5.475	0.87	0.982	0.963	0.585
	P	0.65	**0.039**	**<0.001**	0.366	0.432	0.441	0.695
	η_p_^2^		**0.19**	**0.23**				
12. **FD**	F	2.497	1.094	1.56	0.405	0.704	0.242	0.585
	P	0.135	0.312	0.203	0.534	0.579	0.917	0.679
	η_p_^2^							

S1, session 1; S2, session 2; RET, retention session; G, Group; S, Session; T, Trial; FD, Fractal Dimension. Significant values and their effect sizes, expressed as partial eta squared (η^2^_p_), are indicated in bold.

**Table 2:** Summary of ANOVAs for the one-dimensional parameters (AP, ML).

**S1 vs S2 vs RET**		1. G	2. S	3. T	4. D	5. G x S	6. G x D	7. S x T	8. S x D	9. G x S x D
		df: 1, 18	df: 2, 36	df: 7, 126	df: 1, 18	df: 2, 36	df: 1, 18	df: 14, 252	df: 2, 36	df: 2, 36
1. Angle	F	3.711	40.056	15.147	6.052	3.299	0.334	3.243	0.413	3.267
	P	0.073	**<0**.**001**	**<0**.**001**	**0**.**027**	0.072	0.572	**0**.**003**	0.632	0.062
	η_p_^2^		**0**.**69**	**0**.**46**	**0**.**25**			**0**.**15**		
2. Sway Path	F	0.022	23.669	15.163	0.502	1.311	0.062	3.717	0.961	0.211
	P	0.885	**<0**.**001**	**<0**.**001**	0.49	0.28	0.807	**0**.**002**	0.387	0.788
	η_p_^2^		**0**.**57**	**0**.**46**				**0**.**17**		
3. RMS	F	5.327	36.942	14.695	11.175	3.108	2.056	3.185	0.683	3.026
	P	**0**.**036**	**<0**.**001**	**<0**.**001**	**0**.**004**	0.079	0.172	**0**.**004**	0.486	0.076
	η_p_^2^	**0**.**23**	**0**.**67**	**0**.**45**	**0**.**38**			**0**.**15**		
4. ApEn	F	2.096	8.834	1.643	0.006	2.308	2.601	0.99	4.605	5.356
	P	0.168	**0**.**001**	0.174	0.939	0.121	0.128	0.439	**0**.**028**	**0**.**018**
	η_p_^2^		**0**.**33**						**0**.**20**	**0**.**23**
5. MPF	F	2.384	7.564	1.486	0.777	2.067	1.613	1.872	2.142	2.742
	P	0.143	**0**.**002**	0.223	0.392	0.144	0.223	0.087	0.143	0.09
	η_p_^2^		**0**.**30**							
**S1 vs S2**		**df: 1, 18**	**df: 1, 18**	**df: 7, 126**	**df: 1, 18**	**df: 1, 18**	**df: 1, 18**	**df: 7, 126**	**df: 1, 18**	**df: 1, 18**
6. Angle	F	2.927	90.77	15.375	6.085	13.633	0.008	4.966	0.293	4.816
	P	0.108	**<0**.**001**	**<0**.**001**	**0**.**026**	**0**.**002**	0.928	**0**.**001**	0.596	**0**.**044**
	η_p_^2^		**0**.**80**	**0**.**46**	**0**.**22**	**0**.**38**		**0**.**22**		**0**.**18**
7. Sway Path	F	0.115	46.809	19.074	0.117	0.726	0.093	4.712	1.081	0.403
	P	0.74	**<0**.**001**	**<0**.**001**	0.737	0.407	0.764	**0**.**002**	0.315	0.535
	η_p_^2^		**0**.**68**	**0**.**51**				**0**.**21**		
8. RMS	F	4.058	63.644	16.198	9.369	8.373	0.692	5.11	2.515	6.417
	P	0.062	**<0**.**001**	**<0**.**001**	**0**.**008**	**0**.**011**	0.419	**0**.**001**	0.134	**0**.**023**
	η_p_^2^		**0**.**74**	**0**.**47**	**0**.**30**	**0**.**28**		**0**.**22**		**0**.**23**
9. ApEn	F	1.402	4.832	0.988	0.537	4.004	1.148	1.504	0.514	5.247
	P	0.255	**0**.**044**	0.42	0.475	0.064	0.301	0.201	0.485	**0**.**037**
	η_p_^2^		**0**.**18**							**0**.**23**
10. MPF	F	1.775	3.439	0.896	2.754	3.225	0.897	2.238	0.547	4.626
	P	0.203	0.083	0.473	0.118	0.093	0.359	0.067	0.471	**0**.**048**
	η_p_^2^									**0**.**17**
**S2 vs RET**		**df: 1, 18**	**df: 1, 18**	**df: 7, 126**	**df: 1, 18**	**df: 1, 18**	**df: 1, 18**	**df: 7, 126**	**df: 1, 18**	**df: 1, 18**
11. Angle	F	5.586	8.629	6.23	6.812	0.021	2.477	1.384	0.852	0.537
	P	**0**.**032**	**0**.**01**	**<0**.**001**	**0**.**02**	0.886	0.136	0.243	0.371	0.475
	η_p_^2^	**0**.**20**	**0**.**28**	**0**.**26**	**0**.**24**					
12. Sway Path	F	0.213	4.881	5.419	0.31	0.837	0.001	0.909	2.186	0.001
	P	0.651	**0**.**043**	**<0**.**001**	0.585	0.375	0.97	0.471	0.16	0.972
	η_p_^2^		**0**.**18**	**0**.**23**						
13. RMS	F	7.165	10.337	6.35	14.834	0.288	5.407	1.263	0.158	0.204
	P	**0**.**017**	**0**.**006**	**<0**.**001**	**0**.**002**	0.599	**0**.**035**	0.291	0.697	0.658
	η_p_^2^	**0**.**25**	**0**.**32**	**0**.**26**	**0**.**40**		**0**.**20**			
14. ApEn	F	3.02	3.9	2.268	0.28	0.01	4.32	0.278	10.23	0.44
	P	0.103	0.067	0.067	0.602	0.912	0.055	0.91	**0**.**006**	0.518
	η_p_^2^								**0**.**32**	
15. MPF	F	3.091	3.973	2.36	0.016	0.026	3.346	1.149	2.616	0.534
	P	0.099	0.065	0.075	0.9	0.874	0.087	0.537	0.127	0.476
	η_p_^2^									

S1, session 1; S2, session 2; RET, retention session; G, Group; S, Session; D, Direction;; T, Trial; RMS, Root Mean Square; ApEn, Approximate Entropy; MPF, Mean Power Frequency. Interaction factors with no statistical significance have been omitted. Significant values and their effect sizes, expressed as partial eta squared (η^2^_p_), are indicated in bold.

**Figure 3:**
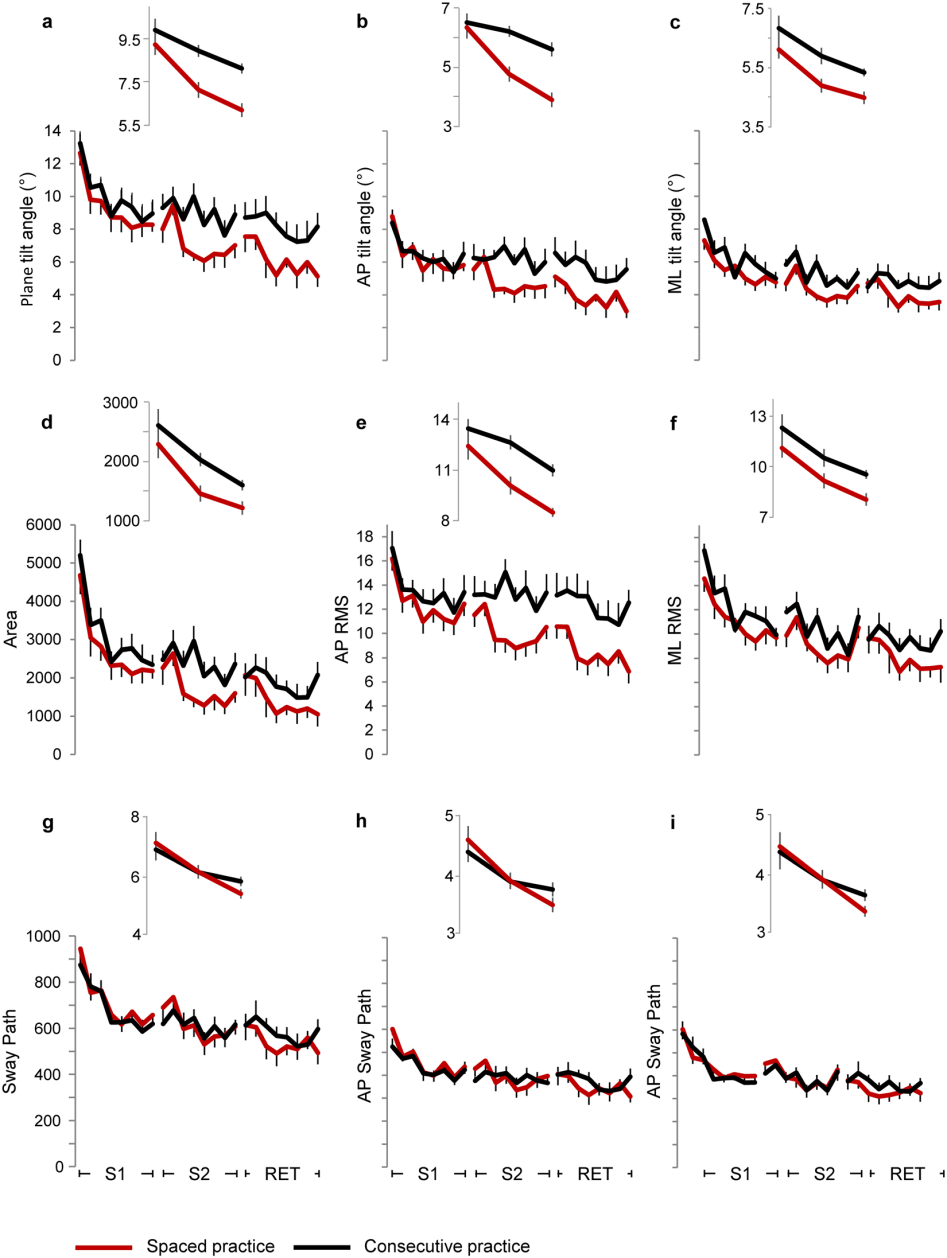
Changes in stability-related parameters. Amplitudes of the tilt board angle around the horizontal plane for any direction (**a**), for the AP direction (**b**), and for the ML direction (**c**). Displacements of the board motion over the horizontal plane represented as Area of sway (**d**), RMS in the AP (**e**) and ML (**f**) direction, total length of sway path (**g**), and sway path along the AP (**h**) and ML (**i**) di**r**ection. The displacement parameters are expressed as unit normal vectors. Each data point represents the grand average over participants performing concentrate (black lines) and spaced (red lines) practice. The error bars represent the standard errors. Abbreviations as in Fig. 2.

**Figure 4:**
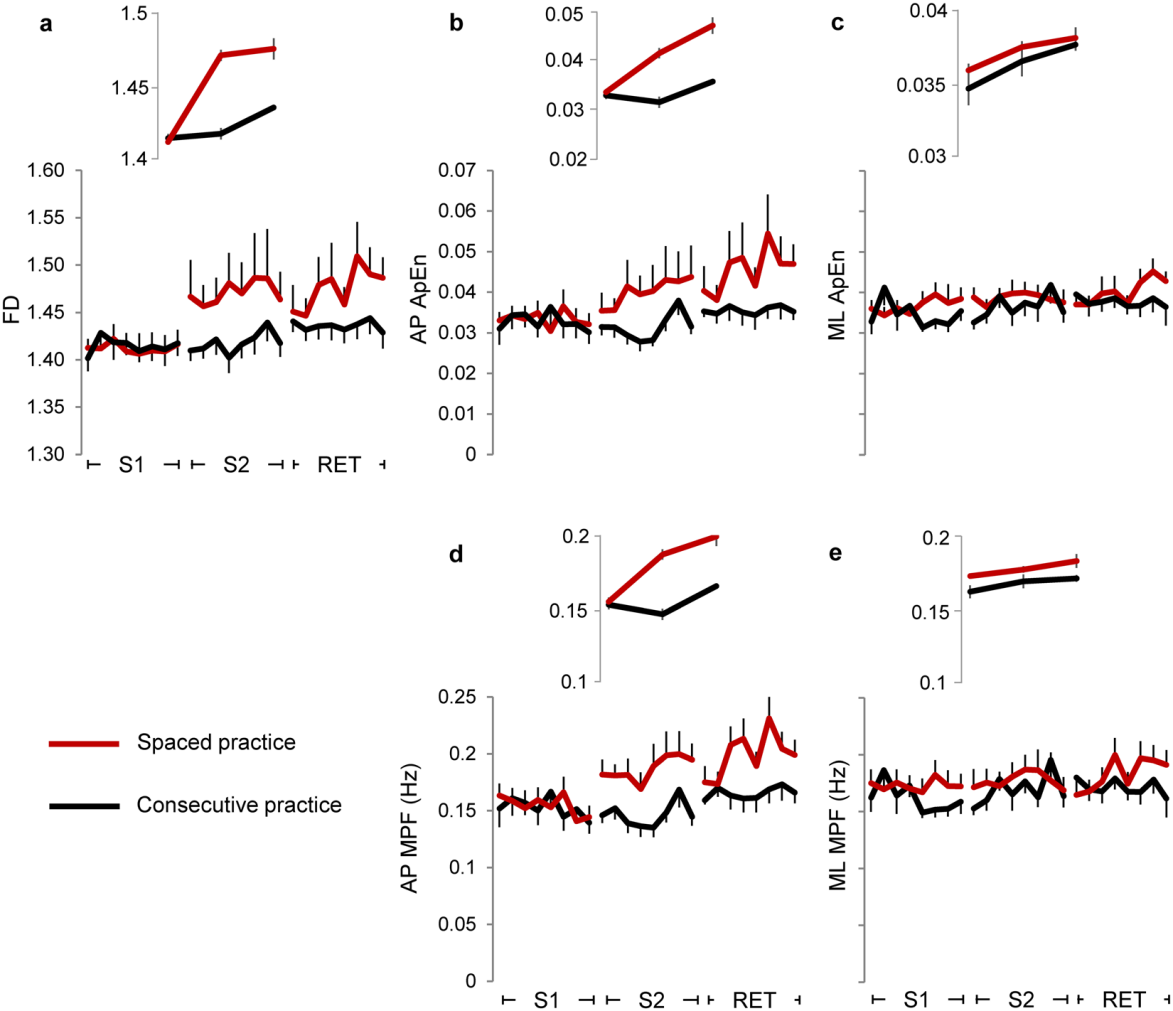
Changes in structure-related parameters. The fractal dimension (FD; **a**) was computed from the trajectory of the board normal vector. The Approximate entropy (ApEn; **b**–**c**) and the Mean Power frequency (MPF; **d**–**e**) were computed from the time series of the board normal vector along AP and ML direction. Symbols and abbreviations as in Fig. 3.

The overall changes in learning and retention across stability-related parameters (Fig. 3) showed significant effects of session, trial and their interaction factor (Table 1: columns 2,3,6, rows 1–3; Table 2: columns 2,3,7, rows 1–3), indicating changes in performance between- and within-session. For the three structure-related parameters (Fig. 3) there was only a main effect of session (Table 1: column 2, row 4; Table 2: column 2, rows 4, 5). Among the parameters associated with the sway direction only the angle of inclination (Table 2: column 4, row 1; Fig. 3b,c) and the root mean square (RMS; Table 2: column 4, row 3; Fig. 3e,f) showed significant changes between the two directions, and only the ApEn (Fig. 4b,c) showed significant interaction of session × direction (Table 2: column 8, row 4) and group × session × direction (Table 2: column 9, row 4). The RMS was the only parameter exhibiting a significant effect of group (Table 2: column 1, row 3; Fig. 3e,f).

The behavior observed along the sessions reflects the overall efficacy of the training in determining learning and consolidation of the postural skill, with a directional bias in the performance improvement.

### Comparison between training sessions

Most of the changes reported over the three sessions occurred passing from S1 to S2. In fact, while there was no main effect of group factor across all of the parameters (Table 1: column 1, rows 5–8; Table 2: column 1, rows 6–10), the stability-related parameters showed important statistical differences and large effect sizes for the session and trial factors (Table 1: columns 2, 3, rows 5–7; Table 2: columns 2, 3, rows 6–8; Fig. 3a-i).

Significant changes between the two sessions occurred for the FD (Table 1: column 2, row 8; Fig. 4a) and the ApEn (Table 2: column 2, row 9; Fig. 4b,c), but the level of significance and the effect size was lower than stability-related parameters. No main effects were reported across the structure-related parameters for the trial factor (Table 1: column 3, row 8; Table 2: column 3, rows 9, 10).

**Figure 5:**
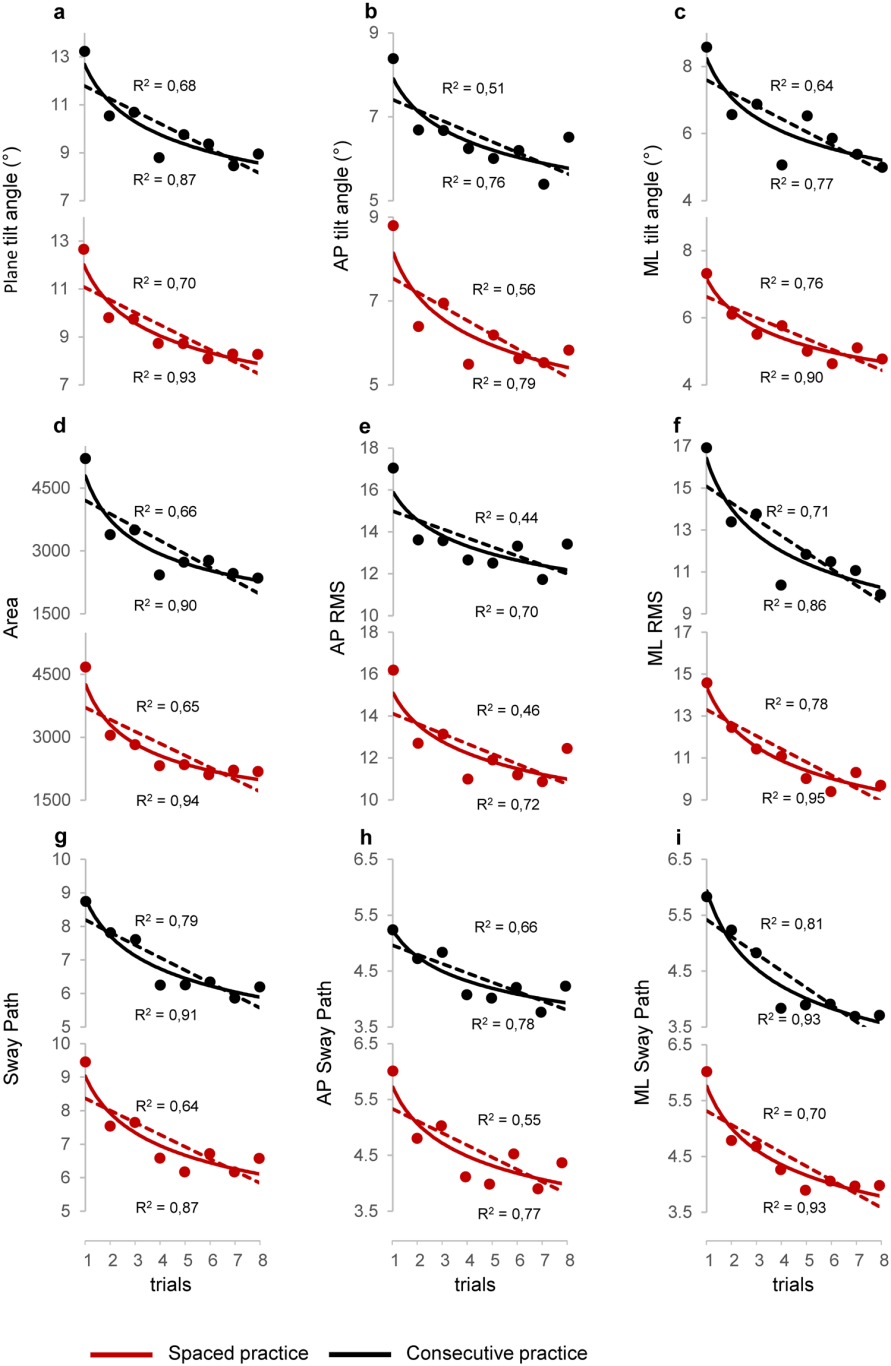
Regression analysis for stability-related parameters in the first session. Linear (dotted lines) and power (solid lines) functions fit to real data (filled circles) observed for each trial in concentrate (black lines) and spaced (red lines) practice. In each plot is reported the coefficient of determination (R^2^) for the linear function (top in the plot) and for the power function (bottom in the plot). Abbreviations and units as in Fig. 3.

While the structure-related parameters were unchanged over the S1 (Fig. 4a–e), the values of the stability-related parameters decrease progressively (Fig. 3a–i). To evaluate the level of saturation of these variations, we fitted linear and power function to the data over the eight trials (Table 3; Fig. 5). The power function exhibited a stronger correlation with the observed data than the linear function, with coefficient of determination (R^2^) ranging from 0.7 to 0.95 for the power function and from 0.44 to 0.81 for the linear function. Moreover, the statistical results reported in Table 3 (much lower sum of squared error (SSE) and root mean squared error (RMSE) for power than linear model) indicate that the power function fitted the data with a level of statistical significance better than the linear model. Thus, in both groups the performance achieved a good level of stability at the end of the S1.

**Table 3:** Model fitting analysis.

		Linear function			Power function		
		R^2^	SSE	RMSE	R^2^	SSE	RMSE
Plane tilt angle	CP	0.68	5.40	0.95	**0**.**87**	2.21	0.61
	SP	0.70	4.78	0.89	**0**.**93**	1.15	0.44
AP tilt angle	CP	0.51	2.56	0.65	**0**.**76**	1.29	0.46
	SP	0.56	3.80	0.80	**0**.**79**	1.79	0.55
ML tilt angle	CP	0.64	3.54	0.77	**0**.**77**	2.24	0.61
	SP	0.76	1.32	0.47	**0**.**90**	0.44	0.27
Area	CP	0.66	2.2E + 06	601	**0**.**90**	6.4E + 05	326
	SP	0.65	1.8E + 06	551	**0**.**94**	3.1E + 05	228
AP RMS	CP	0.44	9.87	1.28	**0**.**70**	5.22	0.93
	SP	0.46	11.39	1.38	**0**.**72**	5.86	0.99
ML RMS	CP	0.71	10.52	1.32	**0**.**86**	5.03	0.92
	SP	0.78	4.61	0.88	**0**.**95**	1.03	0.41
Sway Path	CP	0.79	1.58	0.51	**0**.**91**	0.67	0.33
	SP	0.64	3.01	0.71	**0**.**87**	1.09	0.43
AP Sway Path	CP	0.66	0.59	0.31	**0**.**78**	0.37	0.25
	SP	0.55	1.51	0.50	**0**.**77**	0.78	0.36
ML Sway Path	CP	0.81	0.91	0.39	**0**.**93**	0.34	0.24
	SP	0.70	1.05	0.42	**0**.**93**	0.24	0.20

R^2^, coefficient of determination; SSE, sum of squared error; RMSE, root mean squared error; CP, consecutive practice; SP, spaced practice. Stronger correlations are indicated in bold.

The influence of the pause on the learning process is revealed by the main effect of the interaction of group × session. This interaction was significant for the plane tilt angle and for the area covered by the normal vector of the balance board plane (Table 1: column 4, rows 5, 6), with much more improvements in the SP than in CP group (Fig. 3a,d). No differences were observed for the total length of the sway path (Table 1: column 4, row 7; Fig. 3g). For the one-dimensional parameters, significant interactions of group × session and group × session × direction were observed for the axis tilt angle and for the overall variability measured as RMS (Table 2: columns 5, 9, rows 6, 8), with a larger gap between the groups for the AP than ML direction (Fig. 3b,c,e,f). In addition, the axis tilt angle and the RMS exhibited a main effect of direction, with the values of the AP higher than ML direction (Table 2: column 4, rows 6, 8; Fig. 3b,c,e,f).

The pattern displayed by the trial factor on the stability-related parameters was the same reported for the analysis over all the sessions, with the main effect of trial and the interaction session × trial significant for the plane tilt angle, the area and the sway path (Table 1: columns 3, 6, rows 5–7).

The interaction of group × session was significant only for the FD (Table 1: column 4, row 8; Fig. 4a), but ApEn and MPF showed a significant effect of group × session × direction (Table 2: column 9, rows 9, 10; Fig. 4b–e). For these two structure-related parameters the gap between the groups was larger in the AP than ML direction (Fig. 4b–e).

Again, the effects of trial replicated the results reported when comparing all the sessions, with no significant differences for the three structure-related parameters.

All of these comparisons showed smaller effect sizes with respect to the effect sizes observed for the session factor.

Overall, except for the sway path, subjects performing SP showed significant statistical differences when passing from S1 to S2 with respect to the group executing CP (interaction group × session).For the one-dimensional measurements, the significant changes were limited to the AP direction.

### Estimation of the performance in the retention session

Among the stability-related parameters, the comparison between S2 and RET showed that the main effect of group was observed for the tilt angle in two- and one-dimensional space (Table 1: column 1, row 9; Table 2: column 1, row 11; Fig. 3a–c) and for the RMS (Table 2: column 1, row 13; Fig. 3e,f), while changes in the sway path were not significant for both one- and two-dimensional measures (Table 1: column 1, row 11; Table 2: column 1, row 12; Fig. 3g–i). The sway area was marginally significant (Table 1: column 1, row 10; Fig. 3d), and no significant changes between the groups were observed for all the structure-related parameters (Table 1: column 1, row 12; Table 2: column 1, rows 14, 15; Fig. 4a–e).

The values of all stability-related parameters improved from S2 to RET (main effect of session), with levels of statistical significance and effect sizes lower than those observed between the training sessions (Table 1: column 2, rows 9–11; Table 2: column 2, rows 11–13; Fig. 3a–i). Among the structure-related parameters, the effect of session was marginally significant for the ApEn and MPF (Table 2: column 2, rows 14, 15; Fig. 4b–e).

The statistical results regarding the main effect of trial were consistent with those observed for the comparison between sessions (Table 1: columns 2, 3, rows 9–12; Table 2: columns 2, 3, rows 11–15). However, unlike the comparison between S1 and S2, no significant interaction of session × trial was observed between S2 and RET (Table 1: column 6, rows 9–12; Table 2: column 7, rows 11–15). No other significant change was observed for the factors associated with the trials, but the interaction of group × trial was very close to the level of significance for the tilt angle in two-dimensional space (Table 1: column 5, row 1). This last result reflects the fast performance improvement occurring for the SP group over the trials within S2 and RET.

As for the training sessions, the comparison between S2 and RET showed a main effect of the direction for the axis tilt angle and the RMS, with the values of the AP direction higher than ML direction (Table 2: column 4, rows 11, 13; Fig. 3b,c,e,f).

Among the one-dimensional parameters, the interaction of group × direction was significant for the RMS (Table 2: column 6, row 13; Fig. 4b,c) and the interaction of session × direction was significant for the ApEn (Table 2: column 8, row 14; Fig. 5b,c).

Except for the main effect of the session factor, the effect sizes were comparable with the effect sizes observed for the comparison between training sessions.

These results show that the gain of performance between the two groups observed passing from S1 to S2 was maintained one week after the end of the training. Interestingly, the overall performance continues to improve between and within the S2 and RET (main effects of session and trial) and the marginal significance for the interaction of group × trial is an indication for a possible within-session gain associate with the spaced training.

## Discussion

The results reported in this paper are in line with the idea that spaced practice produces a delayed offline gain with respect to the performance improvements associated with direct training^2,6^.

Two main points can be stressed: first, this study provides a comprehensive quantification of the effects of spaced training on learning a challenging balance task; second, memory processing accomplished during the offline periods leads to specific changes in the spatial and temporal dynamics of the postural sway.

The beneficial effects of post-training intervals reported in the current study replicate the results derived from many experiments using finger or upper limb motor skills^1–3,5,16,18^. Similar findings were also obtained in two studies on learning balancing tasks^21,23^ and in other whole-body motor abilities^23,31–34^.

Although our data indicate that performance improvements associated with offline periods may be shared across motor skills with diverse levels of complexity, the factors modulating this gain could play different roles as the movement demand changes^1,25,35^.

Common elements for the activation of offline memory processing across a variety of motor tasks are the amount of practice and the length and quality of the time interval between the sessions of practice^1,3,9,36^.

### Amount of practice to trigger offline memory processing

The learning based on trial-by-trial error reduction requires a number of repetitions to improve the performance accuracy. However, as the amount of practice increases, learning saturation, instead of the number of trials, becomes the critical factor to trigger both online and offline memory encoding^9,19^.

Over the S1, the performance developed through the typical learning curve, with an early phase of rapid error reduction, followed by a slower decrease to steady state. Except for the structure-related parameters, the performance evolution showed a good fit to the power function, reflecting an appropriate level of learning saturation. Based on the data reported by Hauptmann *et al*.^9^, learning curves following the power function can be associated with the benefits derived from spaced training. We found correlations between learning saturation and the occurrence of offline performance gain for some structure-related parameters (Plane tilt angle, Area, AP tilt angle, AP RMS), but there was no such relation for other parameters (ML tilt angle, ML RMS, Sway Path, FD, ApEn, and MPF). These differences indicate specific sensitivity in encoding the postural parameters during the offline period. In the next sub-sections we will discuss possible explanations for this behavior.

It is noteworthy that during the S2 and RET the performance continued to improve in both groups, with a further gain in the spaced group after the first two trials. Although the typical delayed memory gain shows a homogenous within-session improvement of the performance^1,10,17,18^, a minimum amount of practice was required in our task to recall the novel memory trace and promote the additional gain. This behavior is in accord with the results of Shea *et al*.^22^ that found a similar response testing the effects of spaced learning during one-directional balancing task. A possible interpretation of this result may be provided by the study of Albert and Shadmehr^37^ who demonstrated that current feedback signals may serve as a pattern to teach the motor system how to improve learning. In the case of tasks with multi-joints motion and multi-sensory integration, a rapid within-session retraining of sensory feedback may be particularly important to produce the offline gain.

### Length of offline periods: roles of wakefulness and sleep time

The time spent after the end of training is the other critical factor to trigger offline memory processing. One-day pause included a complete circadian cycle with wakefulness and sleep intervals. Although the issue of how wakefulness and sleep influence offline memory processing is beyond the scope of this study, two elements of our experimental design may provide suggestions on the roles played by wakefulness and sleep intervals in determining the observed postural improvements.

First, as the training was performed in absence of explicit external cues to guide the postural control, subjects adapted and optimized the performance using implicit sensory-motor information. In line with the results reported for sequential finger movements, this feature would imply a contribution of wakefulness period for offline memory encoding^1,5,38,39^. In fact, these authors found that sleep was required for those motor tasks where the performance was guided by explicit external cues, while implicit procedural skills, relying on internal information, benefited from wakefulness intervals. Examples from gross motor skills studies confirm that when an explicit help is provided, the performance improves after a night of sleep but not following wakefulness intervals^23,31,33,40^. Conversely, an effect from time intervals during wakefulness appears when subjects perform complex motor tasks using implicit processes^32,34^.

Second, the subjects were free to select the motion strategy to reach the goal of keeping the balance board in horizontal position. According to several findings, wakefulness and sleep may differentially influence movement strategy and goal accuracy, with the goal elaborated during sleep and the movement strategy during the offline time spent awake^8,17,41^.

### Other factors influencing offline memory processing

Although the critical roles of within-session practice and between-session intervals in triggering the offline processes, other factors may modulate the delayed gain. Some authors reported that providing external feedback, such as the knowledge of results, or encouraging the performance by a reward might be helpful in learning whole-body tasks (see^25,42^). Neither of these conditions were included in our experimental set up, thus changes in these factors did not prevent the occurrence of benefits from the spaced training. However, we cannot exclude that they could modulate the offline gain and provide a further advantage in learning balancing tasks.

### Specific movement components encoded during offline memory processing

In most of the relevant literature, the effects of online and offline processes concern the overall performance improvement. A stimulating finding reported in the current study is that offline processes influenced mainly some components of the postural motion. In fact, as the two directions of sway and all the parameters changed over the trials within the S2 and RET in both the groups (see trial factor in Tables 1 and 2), only some components were sensitive to the between-session pause.

First, benefits from post-training intervals were much stronger for the performance along the AP than ML direction. Second, stability-related parameters (tilt angle amplitude, Area and RMS) improved within the S1 and showed a delayed gain, while significant variations of structure-related parameters (FD, ApEn and MPF) were only associated with offline pauses. Finally, both the parameters representing the geometry of the sway trajectory, i.e Area and Sway path, changed within S1, but only the area covering the board motion exhibited an offline gain.

From a functional perspective, this parcellation may be required to face the high information-processing demand associated with standing balance. In fact, in order to reduce the level of computational complexity and the overall costs during the learning time, the elaboration of motor commands can be broke down into single processing segments^25,43^. The possibility that the optimization of motor learning based on sub-movements handling occurs during offline processes is supported by the study of Pekny and Shadmehr^4^ and Friedman and Korman^18^. Pekny and Shadmehr^4^ found that irrelevant force components, which appear spontaneously during learning to reach in a force field, can be reduced providing a sufficient pause between sessions. On the same line, Friedman and Korman^18^, studying finger opposition sequences, found that timing and movement kinematics contributed in different ways when an interfering task disrupted offline memory encoding.

Although our balancing task consists of continuous movements, the directional components, the stability- and structure-related parameters, and the geometrical parameters exhibit physical and functional properties that should allow separate processing of the individual components.

### Spaced practice improves specifically the movements along the anterior-posterior direction

Changes in the AP direction are physically uncorrelated with changes in the ML direction. Furthermore, joints and muscles controlling sway motion along each direction produce distinct patterns of movement. Given the anatomical constrains of ankle and knee joints, the control of motion along ML direction is limited to hip joint rotations, while the movements in AP direction depend on combinations of ankle, knee, and hip rotations^27^. This morpho-functional asymmetry might explain the specific contribution of the AP direction to the delayed gain. In fact, offline memory encoding could meet the amount of time and computational resources required by the high number of degrees of freedom involved in the AP motion. In this way, the online practice and the related metabolic costs can be reduced after the pause.

The specificity showed by AP direction in the current study appears in conflict with the results reported by Shea *et al*.^22^. These authors, studying the effects of spaced practice when balancing along the ML direction, found that postural performance improved after a pause of one day, with a further gain during the retention test. However, in their study the level of complexity was much lower than that required in our experiments. In fact, the task was accomplished using a single axis balance board and the training was assisted by an explicit visual feedback. To reconcile the results of Shea *et al*.^22^ with our data, we suggest that the level of complexity associated with one-directional balancing is sufficient to activate the offline processing, but as the number of directions increases, the offline memory encoding focuses selectively on the more complex directional component. The slight gap between the groups in the RET, regarding the ML direction, suggests that additional sessions of training could led to significant differences also for this direction.

### Changes in movement dynamics occur only after the pause

The different behavior reported for the stability- and structure-related parameters might depend on the different nature of these two categories of measurements. In fact, reductions of sway amplitude and variability measured by stability-related parameters (angle amplitude, Area, Sway Path, RMS) are directly associated with postural stability improvements. Instead, the structure-related parameters capture patterns of sway motion in the temporal (ApEn), spatial (FD) and frequency (MPF) domains, signaling changes in the motion strategy. Thus, online training during the S1, produced an increase of postural stability, while the form and the frequency of oscillations were unchanged. Structure-related parameters increased in the S2 and RET, indicating a specific offline modulation of the spatio-temporal dynamics and the spectral profile of the postural control (see interactions in Table 1, column 4, row 8, and Table 2, column 10, rows 9, 10).

As the value of ApEn increases, the pattern of oscillations changes from a regular scheme to a more complex temporal dynamics. The same model of behavior can be applied to FD changes, but in relation to the geometrical structure of the motion trajectory. Increasing of temporal and spatial complexity reflects the need to integrate many information for the coordination and optimization of the postural performance. This interpretation is shared with most of the relevant literature^28,29,44–49^.

For example, the loss of ability to produce adaptive and complex motor pattern in many postural disorders is associated with a decrease in ApEn^29,45,49^, while increases in ApEn are reported when postural training is adopted to improve sensory and/or motor coordination in several contexts^28,47,48,50^.

In the same vein, increasing of MPF value might indicate a reorganization of sensory and/or motor coordination. The relationships between changes in spectral profile and production of upright postural strategies are supported by several works reporting that specific spectral profiles can be associated with body oscillation speed^51^, muscular stiffness strategy^52^ and specific visual biofeedback control^53^.

Overall, changes in stability- and structure-related parameters may reflect parallel offline processes where the accuracy of spatial goal is separated by the strategy to achieve that goal. This scheme is in line with the independent elaboration of goal and movement components found out for learning finger sequential movements during offline periods^8,17,18^.

### Sway area decreases after the pause but not the total trajectory length

The values of the area covering the trajectory traced by the board motion and the length of the total sway path are physically uncorrelated. For example, if the area decreases, the sway path can be compacted in a smaller surface, with no changes in the total length. Both the parameters may contribute to the optimization of total motion cost, but a decrease in the area is more directly related to an increase in accuracy of board oscillations. In fact, as the motion of the board normal vector approximates vertical axis, that is, the plane of board approximates the horizontal plane, the area of the vector motion decreases. Thus, while during the S1 the concurrent reduction of the area and the sway path may reflect accuracy and mechanical costs improvements, the offline periods advantage mainly the accuracy enhancement, producing the observed decreases in sway area. It is possible that offline processes contribute to maintain a trade-off between the performance accuracy and the mechanical cost.

In summary, all of these findings strongly support the idea that offline memory consolidation has a more intricate structure than how described up to now. Parceled offline memory processing at several levels of movement control may contribute to optimize the whole performance in relation to the task complexity.

### Practical implications

Balance boards are common tools used in rehabilitation setting to improve or recovery upright postural abilities. The results of the current study may provide important insights to help programming training protocols and make available more quantitative outcomes to evaluate the performance.

In most protocols, the training is planned in relation to the total amount of practice, with poor attention to the distribution and duration of the pauses. Our data emphasize the importance of the intervals to optimize the practice and minimize the exercise intensity. This could be taken into account especially for elderly persons and patients groups for which an exhaustive exercise is not always a feasible option.

The offline elaboration of sub-components of the motor skill suggests that a complete activation of memory processing can be obtained if tasks with large involvement of body segments and sensory information are included in the rehabilitation programs. Moreover, the asymmetric contribution exhibited by the two directional components should be particularly considered when upright postural skills are trained in patients with laterality disorders, such as persons with hemiplegia or lower extremity amputation.

## Conclusions

Learning upright standing on a multiaxial balance board benefits from an interval of one day between the training sessions. The amount of practice and the interval duration arranged in our experimental protocol were appropriate to activate the offline processes and provide a gain in the performance. In particular, the use of a whole-body balancing task revealed that the time spent between the training sessions is used to encode memories associated with specific components of the postural skill. The directional axes of motion, the stability- and structure-related parameters, and the area and the length of sway path, appear to be processed individually during offline memory processing. This parcellation of memory representation should help to face the intrinsic sensory-motor complexity of balancing tasks, optimizing the accuracy and the mechanical cost of the performance.

We believe that these results should stimulate the use of common everyday motor tasks in order to explore more deeply the organization of offline memory consolidation in motor learning and to give more insights helping to improve rehabilitative intervention programs.

## Material and Methods

### Participants

At first, twenty-four healthy male adults without any history of neurological diseases, limbs injuries and disturbances affecting balance, were recruited. Only male participants were engaged in order to reduce the inter-subject variability associated with anthropometric differences. Subjects were excluded *a priori* if they had professional experiences in motor performances requiring to train balance ability, such as surfing, ice skating, skiing, skateboarding, snowboarding, martial arts, gymnastics, and ballet. Four out of twenty-four participants interrupted the tests because of interfering activity with the experimental protocol. The remaining twenty subjects completed the study.

The ethical committee of the University of Catania approved the study. The research methods were performed in accordance with relevant guidelines and regulations and all participants signed an informed consent document according to the Declaration of Helsinki.

### Apparatus and procedures

A wooden multiaxial balance board was used in this study (DOMYOS, DECATHLON, Lille, FR; 40 cm in diameter, 1.5 cm in thickness, and 7 cm in height; Fig. 1a).

The participants were told to stand on the board and trying to keep it as horizontal as possible. Neither visual cues nor explicit instructions were provided to guide the performance, forcing the participants to identify implicitly a successful balancing strategy.

The motion of the balance board was recorded by a 3D optoelectronic system (SMART-D, BTS, Milan, IT). Eight infrared cameras detected signals from eight reflective markers placed on the edge of the board (sampling frequency of 200 Hz).

Feet contour lines traced on the board ensured consistent positioning across the tests, with the center of the subject’s feet matched the center of the board (Fig. 1a). In addition, the board base was marked on the floor to check board translation and correct if necessary.

Prior to the trial initiation, each subject stood on the board supported by two investigators. When the board was horizontal, the investigators left the subject free to start balancing and the trial was recorded for 20 sec. To prevent falls, two investigators were close to the subject during the entire trial. However, all participants completed the trials without falling. Experimental data were collected during 24 trials distributed over three sessions (Fig. 1b). The participants were randomly divided into two groups of ten individuals each. A group performed the training with a short interval of 15 min between the first (S1) and the second (S2) session (consecutive practice, CP), while the other group executed the task with a between-session pause of one day (spaced practice, SP). After one week from the end of training, each group performed an additional session of eight trials to test the level of memory retention and possible further improvements.

The anthropometric measurements from the two groups showed no statistically significant differences: age (years): 24.3 ± 2.5 (CP), 25.9 ± 2.9 (SP), P = 0.21; height (cm): 173.1 ± 5.6 (CP), 174.4 ± 5.4 (SP), P = 0.61; weight (kg): 76.7 ± 8.9 (CP), 73.1 ± 6.1 (SP), P = 0.31.

The distribution of time spent for training and pauses was arranged to prevent the effects of fatigue on the learning process. Trials were interspersed by intervals of 30 sec, achieving a total of 210 sec of rest and 160 sec of exercise for each session. In addition, 15 min of pause between the two sessions in the CP provided additional rest and allowed to verify possible performance impairments due to accumulation of fatigue during the S1. In fact, if the last trials in S1 had been influenced by fatigue, an increase in the performance should have been observed in the first trial of S2, after 15 min of rest^10,54^.

The subjects engaged in the CP were told to relax as possible during the interval of 15 min to prevent possible interferences from physical or cognitive activities. Participants performing SP were asked to follow their normal daily routine during the 24-h interval, avoiding new forms of declarative or procedural learning.

During the recording sessions the participants were barefoot with their eyes focusing on a mark placed on the wall at a distance of 2.5 m. The tests were always carried out at the same time of day, (3 p.m.), in a pleasant room temperature and without interference of external noises.

### Data processing and measurements

Kinematic raw signals from the markers on the balance board were offline interpolated by a cubic spline function and low-pass filtered with a zero-lag second-order Butterworth filter (5-Hz cutoff frequency).

Three out of the eight markers were tracked and used to compute the unit normal vector of the board plane by using the Cartesian equation of a plane. The motion of the balance board was reconstructed from the coordinates of the board normal vector that served to compute tilt angles and spatial displacements of the balance board with respect to the horizontal plane (for the mathematical details see Valle *et al*.^26^). In particular, three angular measurements were performed: absolute value of the angle resulting from any combination of pitch and roll board rotations (plane tilt angle); angle resulting from pitch rotations (AP tilt angle); angle resulting from roll rotations (ML tilt angle). As the values of tilt angles decrease, the board plane approximates the horizontal plane, and the postural performance improves. The representative value of angular changes within a single trial was quantified by computing the mean angle over the data collected during the 20-sec trial (horizontal lines in Fig. 2a–c).

On the basis of the spatial displacement of the board normal vector over the horizontal plane, the following parameters were determined: total area covered by the normal vector computed as the 95% confidence ellipse (Fig. 2d); total length travelled by the normal vector (Sway Path) across the horizontal plane and along the AP (AP Sway Path) and ML (ML Sway Path) direction; root mean square (RMS) calculated for each time series associated with AP (AP RMS; Fig. 2e) and ML (ML RMS; Fig. 2f) direction.

Angular amplitude measures were expressed in degrees, while the spatial displacements in unit normal vector. This set of parameters described the level of stability of the balance board motion (stability-related parameters).

A second set of parameters was arranged to describe the dynamics structure of postural signals in time, space, and frequency domain (structure-related parameters).

The temporal dynamics of the signals along the AP (Fig. 2e) and ML (Fig. 2f) direction was assessed computing the Approximate Entropy (ApEn), while the spatial structure of the planar trajectory of the balance board (Fig. 2d) was evaluated by the Fractal Dimension (FD).

The ApEn determines the likelihood that a pattern repeats over time. The values of the ApEn range between 0 and 2, with a decreasing of regularity in the temporal signal as the value increases. We obtained a single ApEn value for each time series associated with AP (AP ApEn; Fig. 2e) and ML (ML ApEn; Fig. 2f) direction.

The FD evaluates the level of geometrical complexity of a planar trajectory. Two-dimensional FD ranges from 0 to 2, with higher values associated with more complex trajectories.

Power spectral density was performed to explore the frequency domain of the postural sway. The total area of the power spectral density and the Mean Power Frequency (MPF) were calculated from the AP (AP MPF; Fig. 2e) and ML (ML MPF; Fig. 2f) time series. Further details on the computation of non-linear and frequency domain parameters are reported in Valle *et al*.^26^.

All the analyses were performed using a customized MatLab R2012a code (Mathworks, Natick, MA, USA).

### Statistical analysis

Each parameter was quantified from the data collected in each trial. Grand average and variability (Standard Deviation and Standard Error) were computed over the 20 participants, for each of 24 trials, and represented the basis for the statistical analysis and graphic illustrations.

Parametric statistical analysis was adopted after preliminary tests for normality (Shapiro-Wilk test) and for equality of sample variances (Levene’s test) were performed. Changes in postural performance over the learning sessions and the effects of spaced training were analyzed using a three-way Analysis of Variance (ANOVA) for the two-dimensional parameters, and a four-way ANOVA for the one-dimensional parameters. In the three-way ANOVA, session and trial were the within-subjects factor, group was the between-subjects factor and combinations of these factors were the interaction factors. The direction of board motion (AP and ML) was the additional within-subjects factor in the four-way ANOVA.

The F-statistic was adjusted applying Greenhouse-Geisser correction, which produces a p-value more conservative. This procedure is typically applied to the repeated-measures ANOVA to correct the result with respect to a possible violation of the sphericity assumption. The level of statistical significance was set to P < 0.05.

The sample size was determined *a priori* based on the data from our previous paper^26^. Power calculations for an ANOVA with repeated measures and within-between interactions were performed using G-power^55^ (version 3.1.9.2) by effect size specification as in Cohen^56^. Considering the lowest value of effect size reported in Valle *et al*.^26^, a partial eta squared (η^2^_p_) of 0.3 was set as input for the power analysis. A total sample size of 20 participants was required to achieve a power of 0.8, thus, 10 participants for each group were deemed a sufficient quantity for the results to be meaningful.

The effect sizes of the ANOVA outcomes were assessed by using η^2^_p_ that describes the fraction of variance attributed to the independent variables.

For each parameter (P), we used linear ($P=a+b\cdot x$) and power ($P=a\cdot {x}^{-b}$) function to fit the data observed over the eight trials in S1. The coefficient of determination (R^2^), the sum of squared errors (SSE) and the root mean square errors (RMSE) were computed to evaluate which function best fit to the observed data. This analysis provided a quantification of the level of learning stability reached at the end of the S1. A linear best fitting would reflect a little stabilization of the performance, while a power best fitting would be an indication of a progressive learning saturation.

Statistical analysis was performed using SYSTAT, version 11 (Systat Inc., Evanston, IL, USA) and Matlab version R2012a (Mathworks Inc, Natick, MA, USA).

## Electronic supplementary material

Supplementary Table S1
