# Supplementary material for: Selective improvements in balancing associated with offline periods of spaced training
Source: Sci Rep. 2018 May 18;8:7836. doi: 10.1038/s41598-018-26228-4 (PMC5959909; doi:10.1038/s41598-018-26228-4)
Supplement: Supplementary file 1 — Supplementary Table S1 [file 41598_2018_26228_MOESM1_ESM.docx]

**Selective improvements in balancing associated with offline periods of spaced training**

Antonino Casabona, Maria Stella Valle, Carlo Cavallaro, Gabriele Castorina, Matteo Cioni

**Supplementary Table S1**. Measures of variability and frequency of the signals showed as representative examples in Figure 2e, f.

| Parameters | Groups | AP | | | | | ML | | | | |
| --- | --- | --- | --- | --- | --- | --- | --- | --- | --- | --- | --- |
|  |  | S1-T1 | S1-T8 | S2-T1 | S2-T8 | RET-T1 | S1-T1 | S1-T8 | S2-T1 | S2-T8 | RET-T1 |
| RMS | CP | 14.92 | 12.77 | 12.8 | 11.56 | 15.03 | 15.01 | 10.24 | 11.29 | 11.1 | 10.83 |
|  | SP | 17.45 | 15.45 | 11.46 | 6.56 | 7.25 | 13.72 | 8.72 | 12.6 | 11.56 | 11.4 |
| ApEn | CP | 0.034 | 0.042 | 0.036 | 0.031 | 0.024 | 0.037 | 0.039 | 0.044 | 0.039 | 0.036 |
|  | SP | 0.024 | 0.017 | 0.026 | 0.041 | 0.045 | 0.033 | 0.035 | 0.026 | 0.025 | 0.027 |
| MPF (Hz) | CP | 0.165 | 0.168 | 0.148 | 0.145 | 0.138 | 0.167 | 0.161 | 0.163 | 0.177 | 0.175 |
|  | SP | 0.139 | 0.099 | 0.154 | 0.163 | 0.193 | 0.154 | 0.168 | 0.123 | 0.128 | 0.125 |

AP and ML Anterior-Posterior and Medial-Lateral sway oscillations; S, session; T, trial; RET, Retention; RMS, Root Mean Square; ApEn, Approximate Entropy; MPF Mean Power Frequency; CP, Consecutive Practice; SP, Spaced Practice.
